# Microdistribution of Magnetic Resonance Imaging Contrast Agents in Atherosclerotic Plaques Determined by LA-ICP-MS and SR-μXRF Imaging

**DOI:** 10.1007/s11307-020-01563-z

**Published:** 2020-12-07

**Authors:** Yavuz Oguz Uca, David Hallmann, Bernhard Hesse, Christian Seim, Nicola Stolzenburg, Hubertus Pietsch, Jörg Schnorr, Matthias Taupitz

**Affiliations:** 1 Charité - Universitätsmedizin Berlin, corporate member of Freie Universität Berlin, Humboldt-Universität zu Berlin, and Berlin Institute of Health, Charitéplatz 1, 10117 Berlin, Germany; 2 MR and CT Contrast Media Research, Bayer AG, Berlin, Germany; 3 Xploraytion GmbH, Berlin, Germany; 4 European Synchrotron Radiation Facility (ESRF), Grenoble, France; 5 Technische Universität Berlin, Berlin, Germany

**Keywords:** Atherosclerosis, MRI, Gadolinium, Iron oxide nanoparticles, Elemental microscopy, LA-ICP-MS, SXRFS, Extracellular matrix, Arterial calcification

## Abstract

**Purpose:**

Contrast-enhanced magnetic resonance imaging (MRI) has the potential to replace angiographic evaluation of atherosclerosis. While studies have investigated contrast agent (CA) uptake in atherosclerotic plaques, exact CA spatial distribution on a microscale is elusive. The purpose of this study was to investigate the microdistribution of gadolinium (Gd)- and iron (Fe) oxide-based CA in atherosclerotic plaques of New Zealand White rabbits.

**Procedures:**

The study was performed as a *post hoc* analysis of archived tissue specimens obtained in a previous *in vivo* MRI study conducted to investigate signal changes induced by very small superparamagnetic iron oxide nanoparticles (VSOP) and Gd-BOPTA. For analytical discrimination from endogenous Fe, VSOP were doped with europium (Eu) resulting in Eu-VSOP*.* Formalin-fixed arterial specimens were cut into 5-μm serial sections and analyzed by immunohistochemistry (IHC: Movat’s pentachrome, von Kossa, and Alcian blue (pH 1.0) staining, anti-smooth muscle cell actin (anti-SMA), and anti-rabbit macrophage (anti-RAM-11) immunostaining) and elemental microscopy with laser ablation inductively coupled plasma mass spectrometry (LA-ICP-MS) and synchrotron radiation μX-ray fluorescence (SR-μXRF) spectroscopy. Elemental distribution maps of Fe, Eu, Gd, sulfur (S), phosphorus (P), and calcium (Ca) were investigated.

**Results:**

IHC characterized atherosclerotic plaque pathomorphology. Elemental microscopy showed S distribution to match the anatomy of arterial vessel wall layers, while P distribution corresponded well with cellular areas. LA-ICP-MS revealed Gd and Fe with a limit of detection of ~ 0.1 nmol/g and ~ 100 nmol/g, respectively. Eu-positive signal identified VSOP presence in the vessel wall and allowed the comparison of Eu-VSOP and endogenous Fe distribution in tissue sections. Extracellular matrix material correlated with Eu signal intensity, Fe concentration, and maximum Gd concentration. Eu-VSOP were confined to endothelium in early lesions but accumulated in cellular areas in advanced plaques. Gd distribution was homogeneous in healthy arteries but inhomogeneous in early and advanced plaques. SR-μXRF scans at 0.5 μm resolution revealed Gd hotspots with increased P and Ca concentrations at the intimomedial interface, and a size distribution ranging from a few micrometers to submicrometers.

**Conclusions:**

Eu-VSOP and Gd have distinct spatial distributions in atherosclerotic plaques. While Eu-VSOP distribution is more cell-associated and might be used to monitor atherosclerotic plaque progression, Gd distribution indicates arterial calcification and might help in characterizing plaque vulnerability.

**Supplementary Information:**

The online version contains supplementary material available at 10.1007/s11307-020-01563-z.

## Introduction

Atherosclerosis is the major promoter of cardiovascular disease with clinical manifestations including myocardial infarction, ischemic stroke, and sudden death [1]. In clinical routine, arterial stenosis or occlusion is diagnosed by X-ray angiography, computed tomography (CT) angiography (CTA), magnetic resonance imaging (MRI) angiography (MRA), or color-coded sonography (CCS). However, discrimination between stable and vulnerable atherosclerotic plaques is not reliably possible. Smaller plaques, which are not detected by these imaging modalities, can be subject to inflammation-driven complications that might result in fissures or erosions with the risk of thrombotic vessel wall occlusion [2].

Imaging methods including MRI, CT, positron emission tomography (PET), catheter-based virtual histology intravascular ultrasound (VH-IVUS), optical coherence tomography (OCT), and near-infrared spectroscopy (NIRS) are currently being investigated regarding their value for characterizing morphological changes leading to vulnerable plaque [3–7]. Among these methods, contrast-enhanced MRI allows noninvasive angiographic examination and assessment of local plaque composition [8]. Dynamic MRI acquisition after intravenous (IV) injection of contrast agents (CA) offers quantitative characterization of inflammatory processes during plaque progression. Several types of CA have been investigated for these purposes: (I) iron oxide nanoparticles (IONP), which are currently available only for experimental use, and (II) clinically available low-molecular-weight gadolinium (Gd)-based chelates (GBCA).

IONP are extensively used in MR imaging of experimental atherosclerosis particularly for detection of angiogenesis, inflammation, and apoptosis [9, 10]. IONP can be conjugated to antibodies or peptides to serve for targeted visualization of endothelial activity or myocardial infarction [11, 12]. After IV injection, IONP are rapidly cleared by the reticuloendothelial system due to their relatively large size. This provides a unique strategy for contrast-enhanced MRI, since phagocytosis results in concentration of the nanoparticles, which becomes detectable as decreased signal intensity typically 24 h after IV injection [13, 14]. Studies investigating very small superparamagnetic iron oxide nanoparticles (VSOP) with a hydrodynamic diameter of 7 nm, longer blood half-life, and faster vascular distribution due to their citrate surface coating have demonstrated even earlier uptake (< 2 h) into the atherosclerotic plaques, which correlated with the accumulation of extracellular matrix (ECM) [15, 16].

GBCA are known as nonspecific agents that result in increased MRI signal intensity through pronounced perfusion and vascular permeability. Although GBCA were developed to distribute within the vascular and extracellular space, and to be eliminated as intact molecules *via* the kidneys, studies have demonstrated Gd deposition predominantly around vascularized areas of the skin, brain, liver, and kidney [17, 18]. Systemic metal deposition was proposed, and colocalization of elements including phosphorus (P) or calcium (Ca) has suggested transchelation of Gd by physiological anions [19–21]. Although Gd deposition in the heart and aorta has also been reported, quantitative research in atherosclerotic plaques remains inadequate [19].

Discrimination between stable and vulnerable atherosclerotic plaques by contrast-enhanced MRI entails better understanding of IONP or GBCA interactions with plaque ECM components. Thus, elucidating microdistribution of CA in atherosclerotic plaques at different stages of disease progression by an experimental workflow with improved tissue detection ability offers a sophisticated basis. In this regard, mass spectrometry and X-ray fluorescence (XRF) analysis have emerged as two cutting-edge elemental microscopy techniques offering the lowest limit of detection (LOD) for reliable quantification of elements in tissue specimens and imaging characterization at the highest spatial resolution, respectively [22, 23].

In this study, we aimed to investigate the microdistribution of Eu-VSOP and Gd-BOPTA (gadobenate dimeglumine) in atherosclerotic plaques of New Zealand White (NZW) rabbits using immunohistochemistry (IHC) and elemental microscopy by laser ablation inductively coupled plasma mass spectrometry (LA-ICP-MS), and synchrotron radiation μXRF (SR-μXRF) spectroscopy.

## Materials and Methods

### Animal Procedures

The study was conducted in accordance with the requirements of directive 2010/63/EU and the German Animal Protection Act and approved by the local animal protection committee of the Landesamt für Gesundheit und Soziales (LAGeSo, Berlin State Office for Health and Social Affairs, Germany). Experimental conditions were constant at all times (see electronic supplementary material for induction of atherosclerosis). Twelve male NZW rabbits were IV injected with VSOP at a dose of 0.05 mmol Fe/kg body weight. VSOP were synthesized at the Charité Department of Radiology according to the protocol described by de Schellenberger et al. and had the following properties: 0.5 M Fe concentration with 13 % citric acid (weight/weight total Fe), 3 g/l sodium glycerophosphate, 2 g/l N-methylglucamine, and 60 g/l mannitol [24]. Synthesis was adapted to yield the final pharmaceutical formulation of the investigational drug VSOP-C184 used in clinical trials [25]. For unambiguous analytical discrimination from endogenous Fe, VSOP were doped with europium (Eu) by substituting ferric ions in a 5 % weight ratio of Eu3+ to Fe3+ resulting in Eu-VSOP, which has no influence on the magnetic properties of the particles [26]. At 1 h after Eu-VSOP injection, 10 rabbits were IV injected with Gd-BOPTA (Bracco Imaging Deutschland GmbH, Konstanz, Germany) at a dose of 0.1 mmol/kg, since Gd-BOPTA was often preferred for vascular MRI. The rabbits were sacrificed 2 h after the initial CA administration. The vascular system was perfused with electrolyte solution and the aortic arch was removed and processed by formalin fixation at 4 °C overnight and embedded in paraffin. Atherosclerosis-free control specimens (Eu-VSOP-negative and Gd-positive controls were Vasovist and elastin-specific CA (BMS753951) administered at a dose of 0.2 mmol/kg) were kindly provided by Marcus Makowski from our institution and processed under the same conditions [27].

### Immunohistochemistry

The paraffin blocks were cut into 5-μm serial sections using an automated microtome, which were mounted on SuperFrost Ultra Plus microscope slides (VWR International, Geldenaaksebaan, Belgium) for IHC and LA-ICP-MS, or on ultralene foil for SR-μXRF analysis. Movat’s pentachrome, von Kossa, and Alcian blue (pH 1.0) histologic staining, anti-smooth muscle cell (SMC) actin (anti-SMA), and anti-rabbit macrophage (anti-RAM-11) immunostaining procedures were carried out on adjacent sections as described elsewhere [16]. Qualitative and score-based semiquantitative assessment of plaque pathomorphology was done by independent observers recording pathologic features defined by the American Heart Association (AHA) (electronic supplementary material, Table 1) [28]. Digital high-resolution scans of the histologic specimens were obtained at the Zentrale Biomaterialbank der Charité (ZeBanC).

### Laser Ablation Inductively Coupled Plasma Mass Spectrometry

LA-ICP-MS analysis was performed on the sections adjacent to IHC sections. For that, an ICP-MS (Agilent 7900, Waldbronn, Germany) was coupled to a laser ablation system (NWR 213; New Wave Research, California, USA). Prior to analysis, sections were deparaffinized. Laser ablation was performed in continuous-line ablation mode with a circular laser spot size of 20 μm at a scanning speed of 100 μm s^−1^ and 200 ms acquisition time with daily-optimized output energies of 1.5 J cm^−1^. The method has been described elsewhere [29]. Ablated tissue was transported into the ICP-MS imager with helium gas at a flow of 0.9 L/min. Matrix-matched laboratory standards of well-defined element concentrations were spiked onto gelatin to quantify Gd and Fe (see also electronic supplementary material, Fig. S3). Fe concentration was determined by analysis of the isotope at mass 57 due to strong interference of ^40^Ar^16^O with the most abundant isotope at mass 56 [30]. Gd and Eu signals were determined by analysis at mass 158 and 153, respectively. Eu signal was only recorded in terms of counts per second (CPS) due to unavailability of standards. Generation of 2D distribution maps, image processing, and data evaluation were performed using MassImager, a free software developed by Robin Schmidt [31].

### Synchrotron X-Ray Fluorescence Spectroscopy

SR-μXRF investigations on the sections adjacent to those used in LA-ICP-MS analysis were done at the ID21 beamline at the European Synchrotron Radiation Facility in Grenoble, France [32]. Experiments were performed using the in-vacuum scanning X-ray spectroscopy setup, in which X-rays were generated by undulators with an optimized gap size for 7.3 keV. The setup is explained in detail elsewhere [33]. The X-ray beam was focused down to ~ 0.6 × 0.8 μm^2^ (vertical × horizontal) using a fixed-curvature Kirkpatrick-Baez mirror system. Flux was ~ 5 × 10^10^ photons/s (~ 180 mA SR current in multibunch mode). Acquisition time per pixel was 100 ms. Pixel size for collecting the XRF maps was set to 30 μm, 10 μm, 1–2 μm, or 0.5 μm depending on the size of the region of interest (ROI). Scans were acquired in continuous mode. Distribution maps of Fe, Gd, sulfur (S), P, and Ca were generated. XRF normalization, spectral deconvolution, and quantification were done using the PyMCA software (see also electronic supplementary material, Fig. S4) [34]. Cellular uptake of Gd was investigated by analyzing the P distribution as a marker of cell membrane, ATP, or nucleic acids. P distribution maps at 0.5 μm resolution were segmented into two compartments by applying a 25 % threshold on the maximum of the P K-line fluorescence signal and comparing elemental concentrations in P-poor (< 25 % P-Threshold) or P-rich (> 25 % P-Threshold) areas. Corresponding spectral deconvolution displaying colocalizing elements was normalized by the number of pixel of each region, thus giving an averaged spectrum for each region. The amplitude of the peak corresponding to the respective element was scaled by the amount of atoms being probed by the X-ray beam. Size distribution analysis of Gd hotspots was done using the ImageJ software.

## Results

**Fig. 1.**
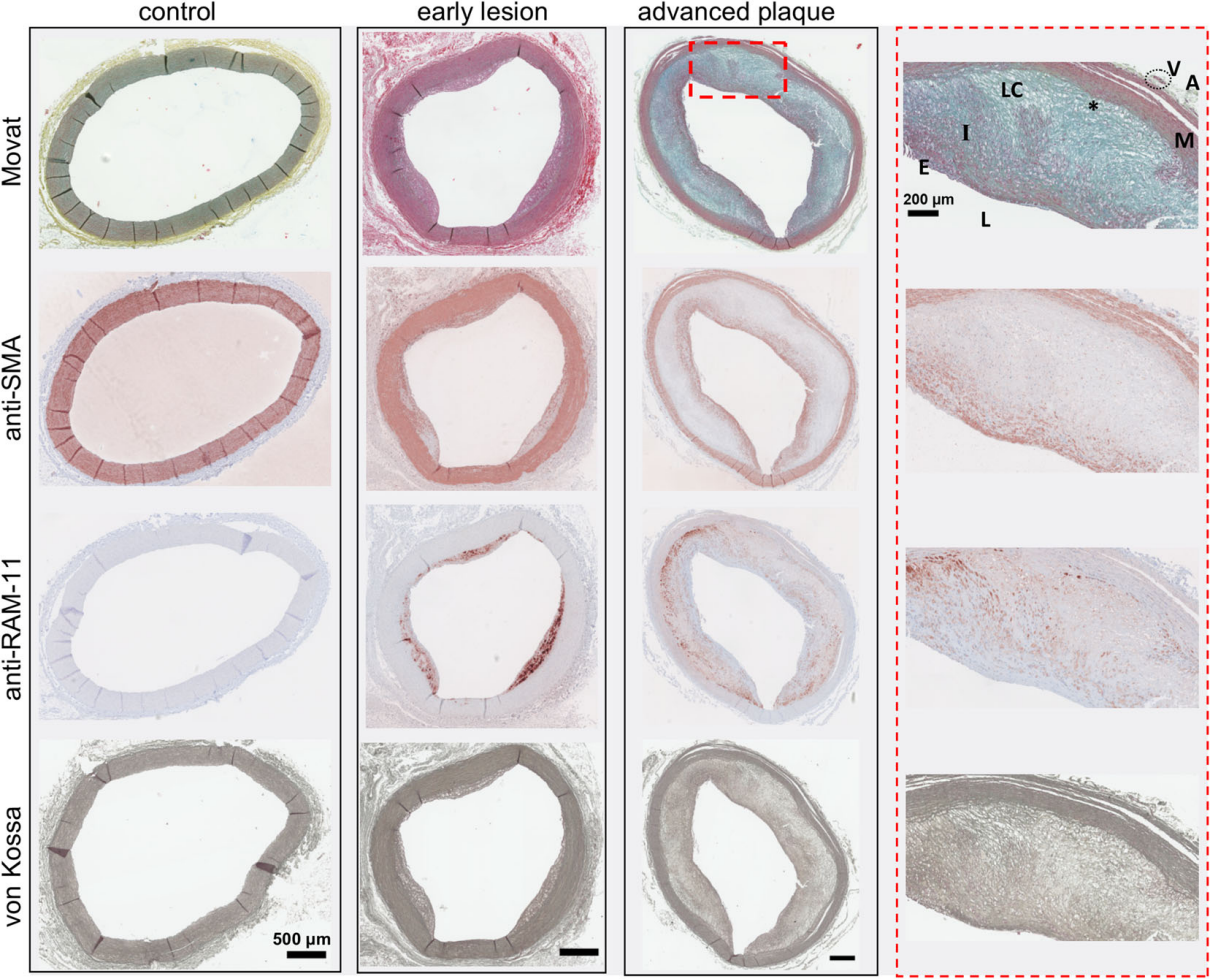
Atherosclerotic plaque characterization by immunohistochemistry (IHC). Advanced plaque region of interest (ROI) (× 10 magnification) is provided for comparison. Movat’s staining reveals cells by red staining that are also depicted by anti-smooth muscle cell (SMC) actin (anti-SMA) and anti-rabbit macrophage (anti-RAM-11) immunostaining. Contractile SMCs in the media and synthetic SMCs migrating into the intima are distinguished by their spindle-like and circular shapes, respectively. Lipid pools are nonstained circular, cleft-, or vacuole-like shapes at the intimomedial areas associated with increased macrophage colocalization, indicating foam cell or lipid cores. Lipid pools are surrounded by light blue-stained glycosaminoglycans (GAG). von Kossa staining identifies calcifications in the intima, especially along the intimomedial interface of advanced plaques. Scale bars, 500 μm; ROI, 200 μm; *L*, lumen; *E*, endothelium; *I*, intima; *LC*, lipid core; asterisk, intimomedial interface; *M*, media; *A*, adventitia; *V*, vasa vasorum.

IHC performed on adjacent sections of arterial specimens obtained from 14 rabbits revealed various degrees of atherosclerotic plaque formation. These were categorized into early lesion and advanced plaque using the pathologic plaque features defined by the AHA (Fig. 1, control: *n* = 2, early lesion: *n* = 3, advanced plaque: *n* = 9, see electronic supplementary material Table 1). Movat’s staining revealed the individual arterial vessel wall layers (adventitia, media, intima, and endothelium)*.* Medial and endothelial regions were the major cellular areas, identified by red staining, which was also depicted by SMA and RAM-11 immunostaining. Contractile SMCs in the media and synthetic SMCs migrating into the intima were distinguishable by their spindle-like and circular shapes, respectively. Large lipid pools were seen as nonstaining circular, cleft-, or vacuole-like shapes mostly at the intimomedial interface associated with increased macrophage colocalization, indicating foam cell or lipid core formation. These were often surrounded by light blue-stained glycosaminoglycan (GAG) networks. Synthetic SMCs and stretches of yellow/green-stained collagen fibrils were observed covering the lipid pools. von Kossa staining revealed intimal calcifications, especially along the intimomedial interface of advanced plaques (see also electronic supplementary material, Fig. S1).

**Fig. 2.**
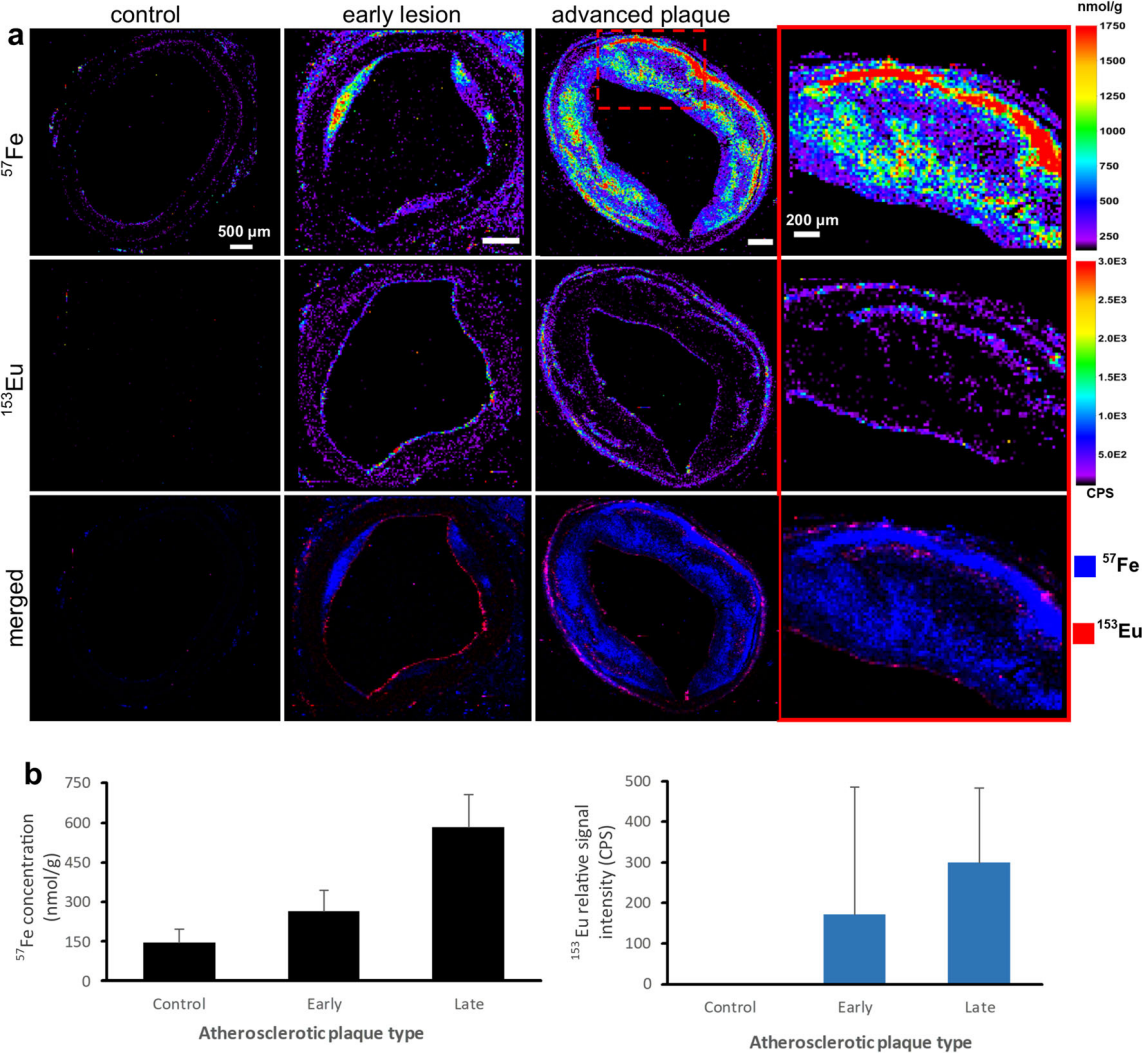
Europium (Eu)-doped very small superparamagnetic iron (Fe) oxide nanoparticle (Eu-VSOP) distribution in atherosclerotic plaques. **a** Laser ablation inductively coupled plasma mass spectrometry (LA-ICP-MS) elemental maps of ^57^Fe^1+^ and ^153^Eu^1+^ distribution. Advanced plaque ROI is provided for comparison. Eu-positive signal (recorded in counts per second, CPS) distinguishes Eu-VSOP distribution from that of endogenous Fe. No Eu signal is detected in controls; weaker Eu signal is detected in the media and the intima of early lesions. Eu signal intensity and Fe concentrations increase in advanced plaques. High Eu signal is confined to endothelium or to the intimomedial interface. Scale bars, 500 μm, ROI, 200 μm. **b** Graphs of Fe concentration and Eu signal intensity in atherosclerotic plaques. Fe concentrations: 146.17 nmol/g (± 52.58 nmol/g) in healthy arteries, 264.89 nmol/g (± 79.23 nmol/g) in early lesions, and 583.20 nmol/g (± 122.46 nmol/g) in advanced plaques.

Metal and nonmetal images obtained by elemental microscopy matched the pathomorphologic features identified by IHC with S distribution highlighting the anatomy of the arterial vessel wall layers, while P distribution corresponded well with cellular areas (Fig. 6). LA-ICP-MS revealed Gd or Fe distribution at 20-μm resolution (*x*-*y* direction) with a LOD of ~ 0.1 nmol/g for Gd, and ~ 100 nmol/g for Fe. No Eu signal was detected in controls (Fig. 2). Eu-positive signal identified the VSOP presence in the vessel wall, and by acting as a surrogate marker, it allowed the comparison of Eu-VSOP and endogenous Fe distribution in tissue sections. Weaker Eu signal was detected in the media and the intima of early lesions. Eu signal intensity and Fe concentrations were higher in larger plaques compared to smaller ones. High Eu signal was confined to endothelium or the intimomedial interface. Fe concentrations were 146.17 nmol/g (± 52.58 nmol/g) in healthy arteries, 264.89 nmol/g (± 79.23 nmol/g) in early lesions, and 583.20 nmol/g (± 122.46 nmol/g) in advanced plaques.

**Fig. 3.**
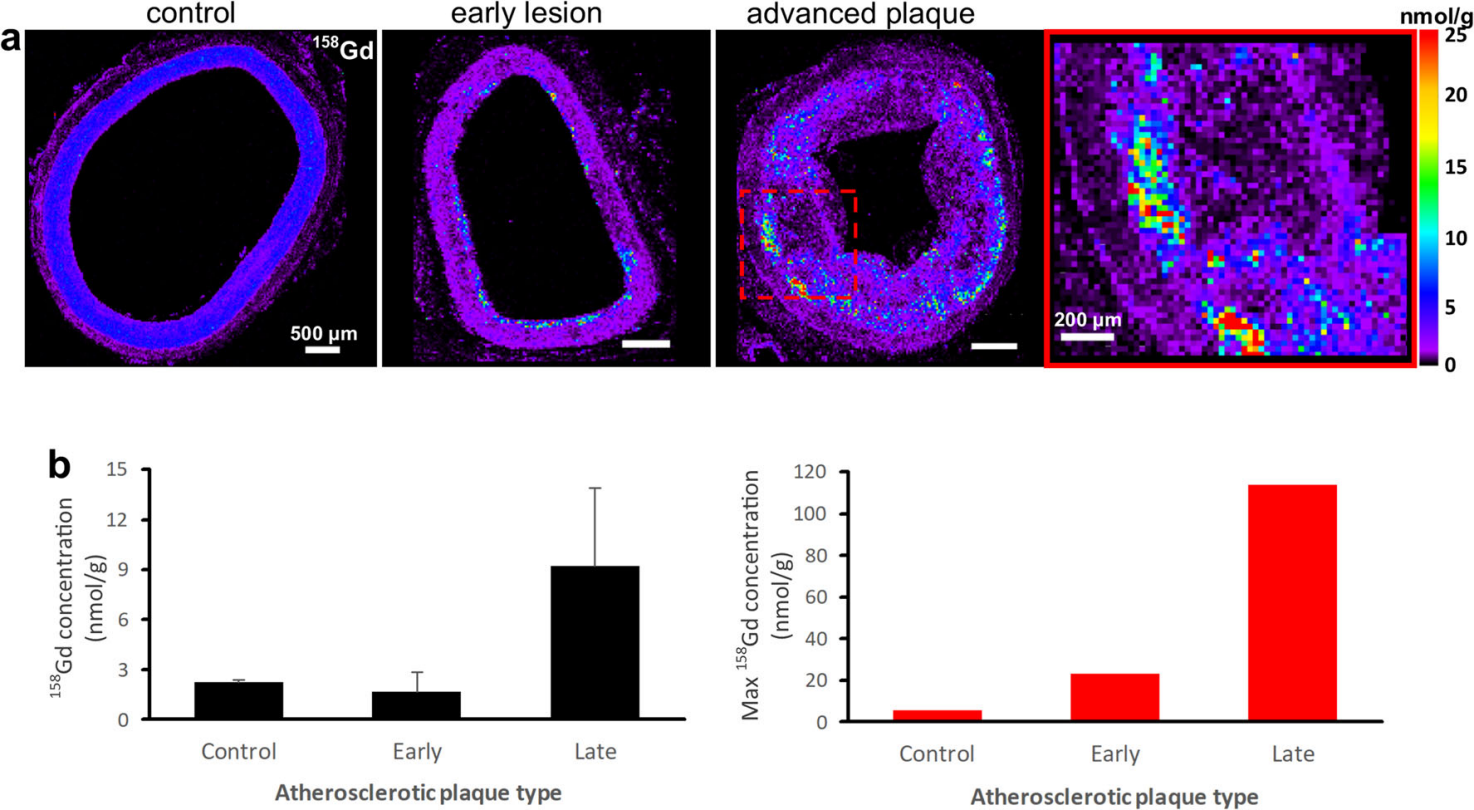
Gadolinium (Gd) distribution in atherosclerotic plaques. **a** LA-ICP-MS elemental map of ^158^Gd distribution. Advanced plaque ROI is provided for comparison. Gd is also detected in the control group (Eu-VSOP-negative, Gd-positive). In healthy arteries, Gd distribution is global and homogenous. In early lesions, Gd concentrations are higher in the subendothelial space. Advanced plaques are characterized by high focal Gd content at the intimomedial interface. Scale bars, 500 μm; ROI, 200 μm. **b** Graphs of Gd and maximum Gd concentration in atherosclerotic plaques. Gd concentrations: 2.22 nmol/g (± 0.14 nmol/g), 1.63 nmol/g (± 1.19 nmol/g), and 9.23 nmol/g (± 4.64 nmol/g), with maximum concentrations of 5.30 nmol/g, 22.85 nmol/g, and 113.66 nmol/g in healthy arteries, early, and advanced plaques, respectively.

In the control group (Eu-VSOP-negative, Gd-positive), Gd was detected (Fig. 3). In healthy arteries, Gd distribution was global and homogeneous. In early lesions, Gd was distributed inhomogeneously and concentrations were higher in the subendothelial space. Advanced plaques were characterized by high focal Gd content at the intimomedial interface. Gd concentrations were 2.22 nmol/g (± 0.14 nmol/g), 1.63 nmol/g (± 1.19 nmol/g), and 9.23 nmol/g (± 4.64 nmol/g), with maximum concentrations of 5.30 nmol/g, 22.85 nmol/g, and 113.66 nmol/g in healthy arteries, early, and advanced plaques, respectively.

**Fig. 4.**
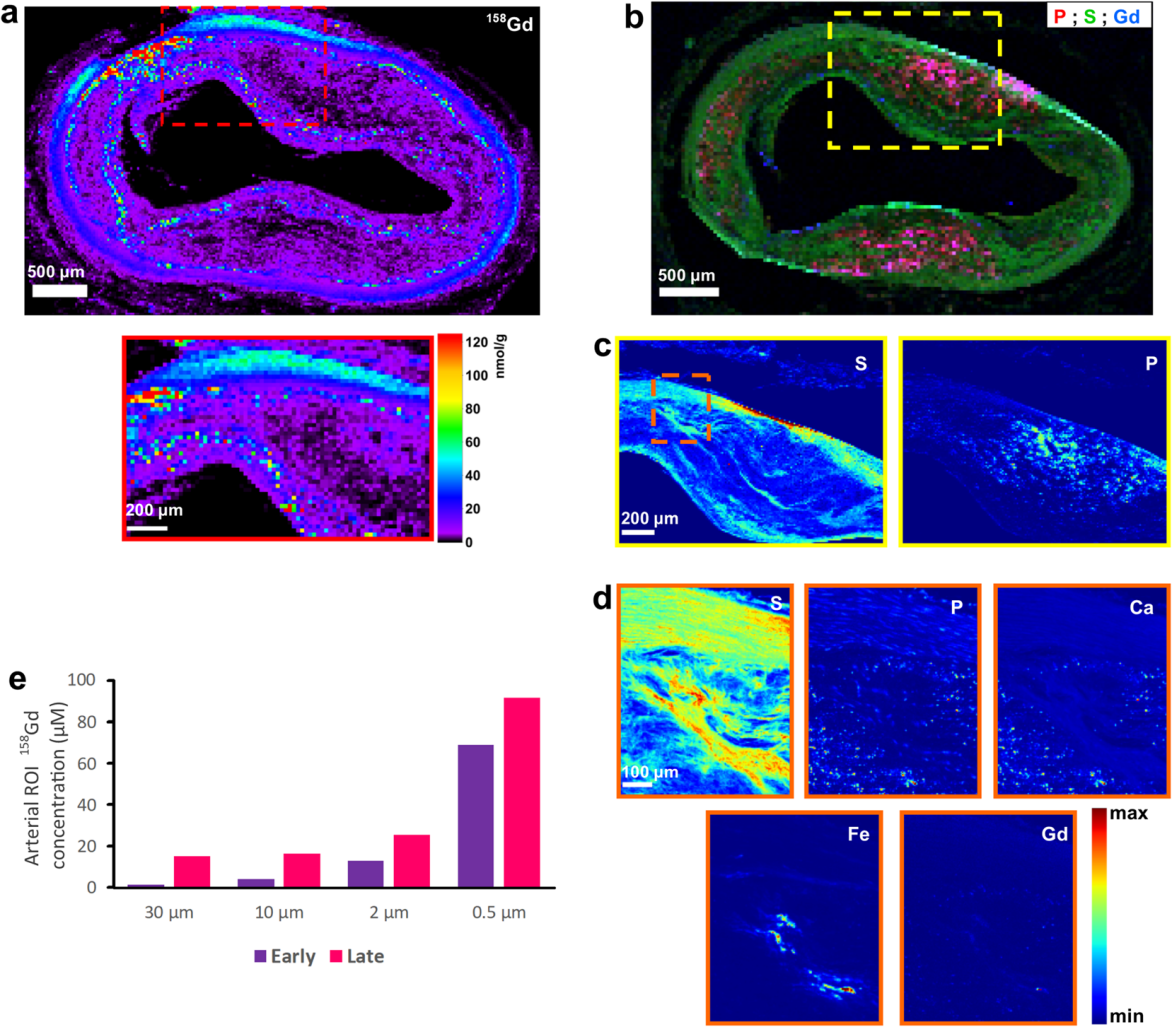
Spatially resolved Gd distribution and elemental hotpots in atherosclerotic plaques. **a** LA-ICP-MS elemental map of ^158^Gd distribution. Advanced plaque ROI is provided for comparison. Scale bars, 500 μm; ROI, 200 μm. **b**–**d** Synchrotron radiation μX-ray fluorescence (SR-μXRF) analysis at increasing resolution. **b** RGB overlay of phosphorus (P), sulfur (S), and Gd distribution at 30-μm resolution. **c** ROI elemental maps of S and P at 10-μm resolution reveal arterial vessel wall anatomy and cellular zones. **d** ROI elemental maps of S, P, calcium (Ca), Fe, and Gd at 2-μm resolution. Scale bars: b1, 500 μm; b2, 200 μm; b3, 50 μm. **e** SR-μXRF analysis in both early lesions and advanced plaques reveals increasing ROI Gd concentrations with increasing spatial resolution.

**Fig. 5.**
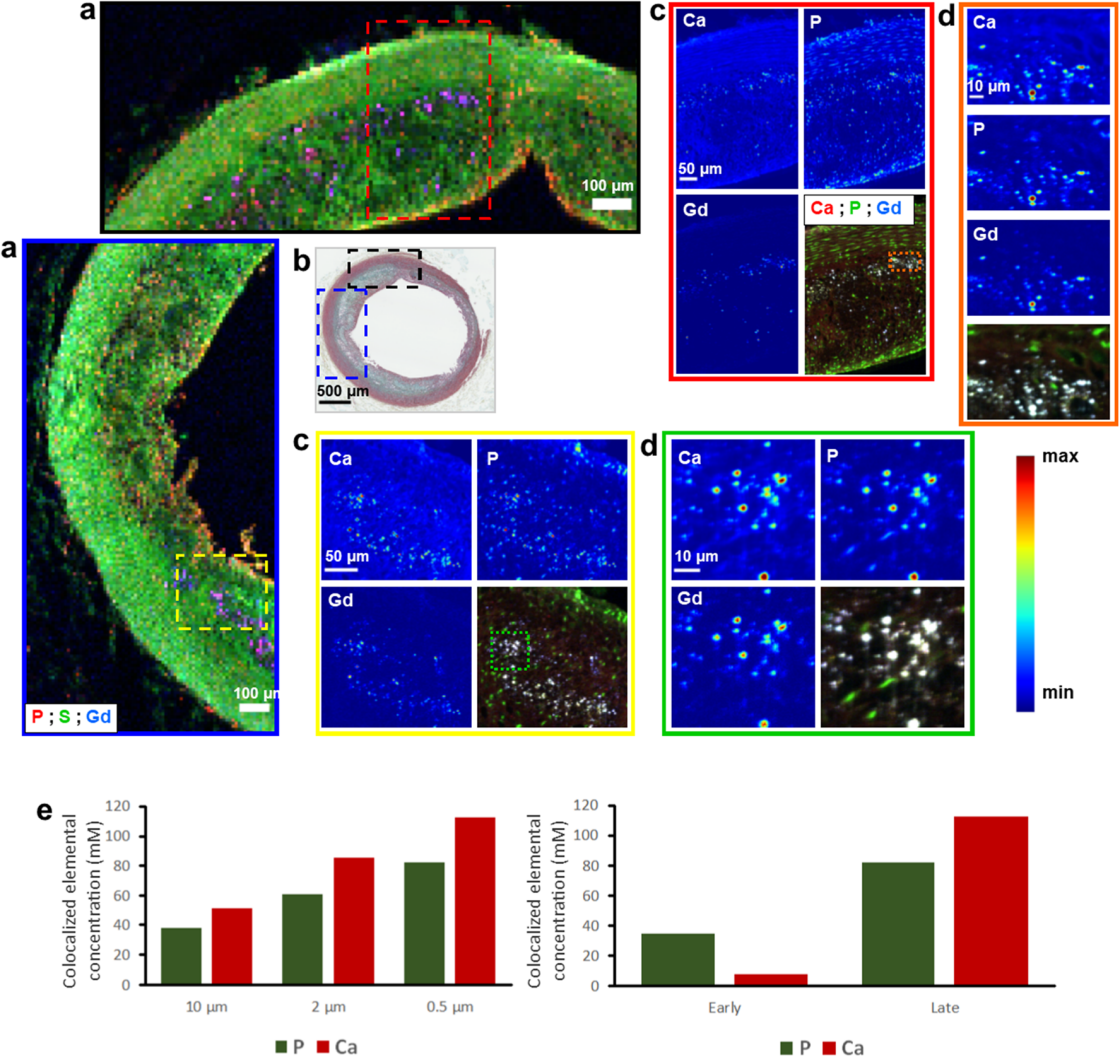
Spatially resolved Gd hotpots and colocalization of P and Ca in atherosclerotic plaques. SR-μXRF analysis results of 2 ROIs in an advanced plaque are provided for comparison. **a** RGB overlay P, S, and Gd distribution at 10-μm resolution. Scale bars, 100 μm. **b** Movat’s staining micrograph is provided for an overview. **c** Elemental maps and RGB overlay of Ca, P, and Gd distribution at 2-μm resolution. Scale bars, 50 μm. **d** Elemental maps and RGB overlay of Ca, P, and Gd distribution at 0.5-μm resolution. Scale bars, 10 μm. **e** SR-μXRF analysis of Gd hotspots reveals higher P and Ca concentrations in progressing plaques (*right*), and concentrations further increase with increasing spatial resolution (*left*).

**Fig. 6.**
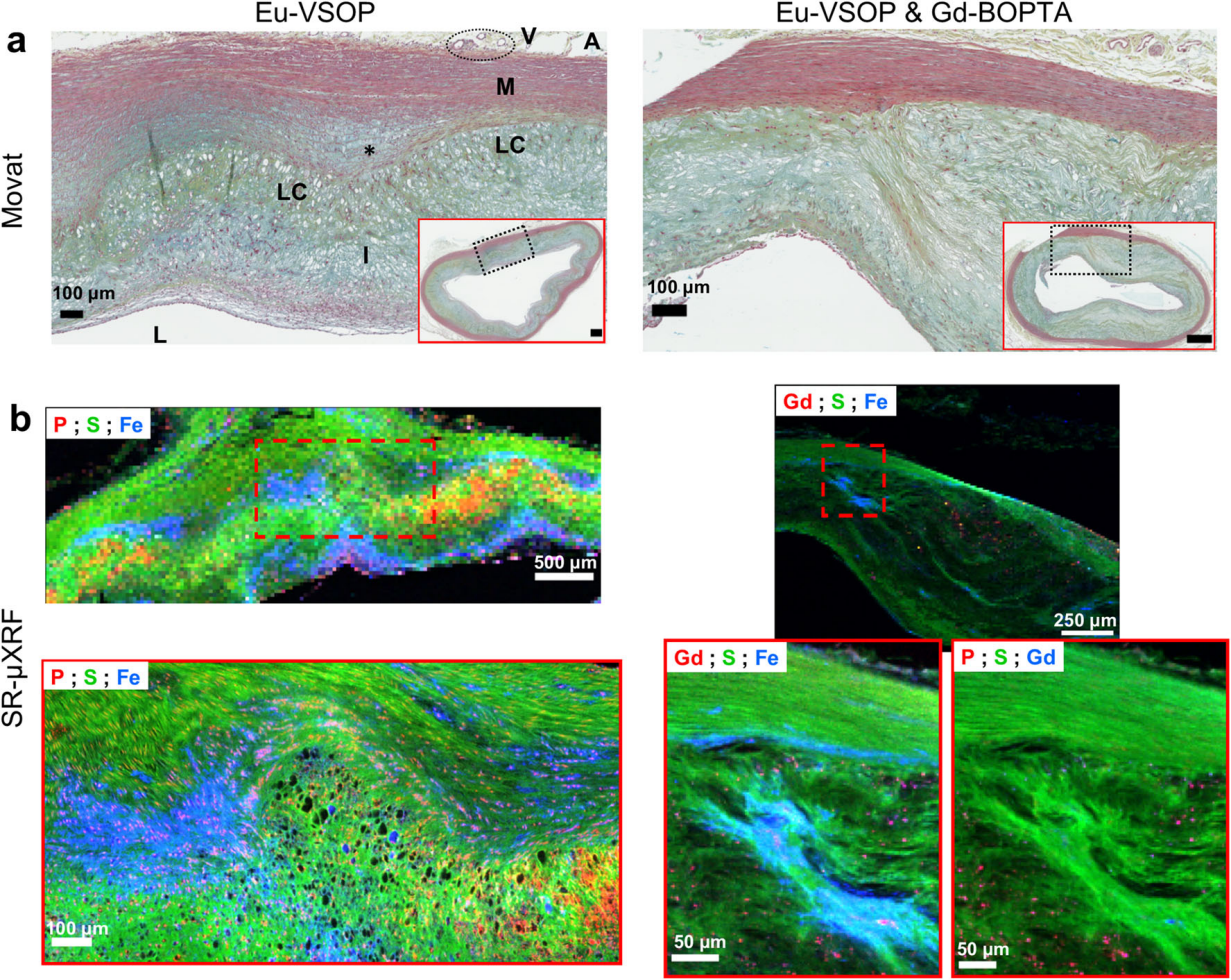
Comparative microdistribution of Fe and Gd in atherosclerotic plaques. Rabbits were intravenously injected with either Eu-VSOP alone (*left*) or Eu-VSOP and Gd-BOPTA (*right*). **a** Microscopic characterization of Movat’s stained advanced plaque pathomorphology. (ROIs, × 10 magnification). Synthetic SMCs and dense stretches of yellow/green-stained collagen fibrils are seen covering the lipid pools. Scale bars, 500 μm; ROI, 100 μm. **b** SR-μXRF analysis at 10-μm and 2-μm resolution (ROIs are indicated by red dashed lines). RGB overlay of P, S, and Fe maps demonstrates distinct Fe distribution in cell- or collagen-rich areas in the intima or at the boundary between the media and intima (*left*; scale bars, 500 μm; ROI, 100 μm). RGB overlay of Gd, S, and Fe maps in addition to P, S, and Gd maps revealed Gd distribution within the deeper areas of the intima, especially along the intimomedial interface at highly increasing concentrations colocalized with P and Ca, distinguishing Gd distribution from that of Eu-VSOP (*right*; scale bars, 250 μm; ROI, 50 μm). *L*, lumen; *I*, intima; *LC*, lipid core; asterisk, intimomedial interface; *M*, media; ***A***, adventitia; ***V***, vasa vasorum.

**Fig. 7.**
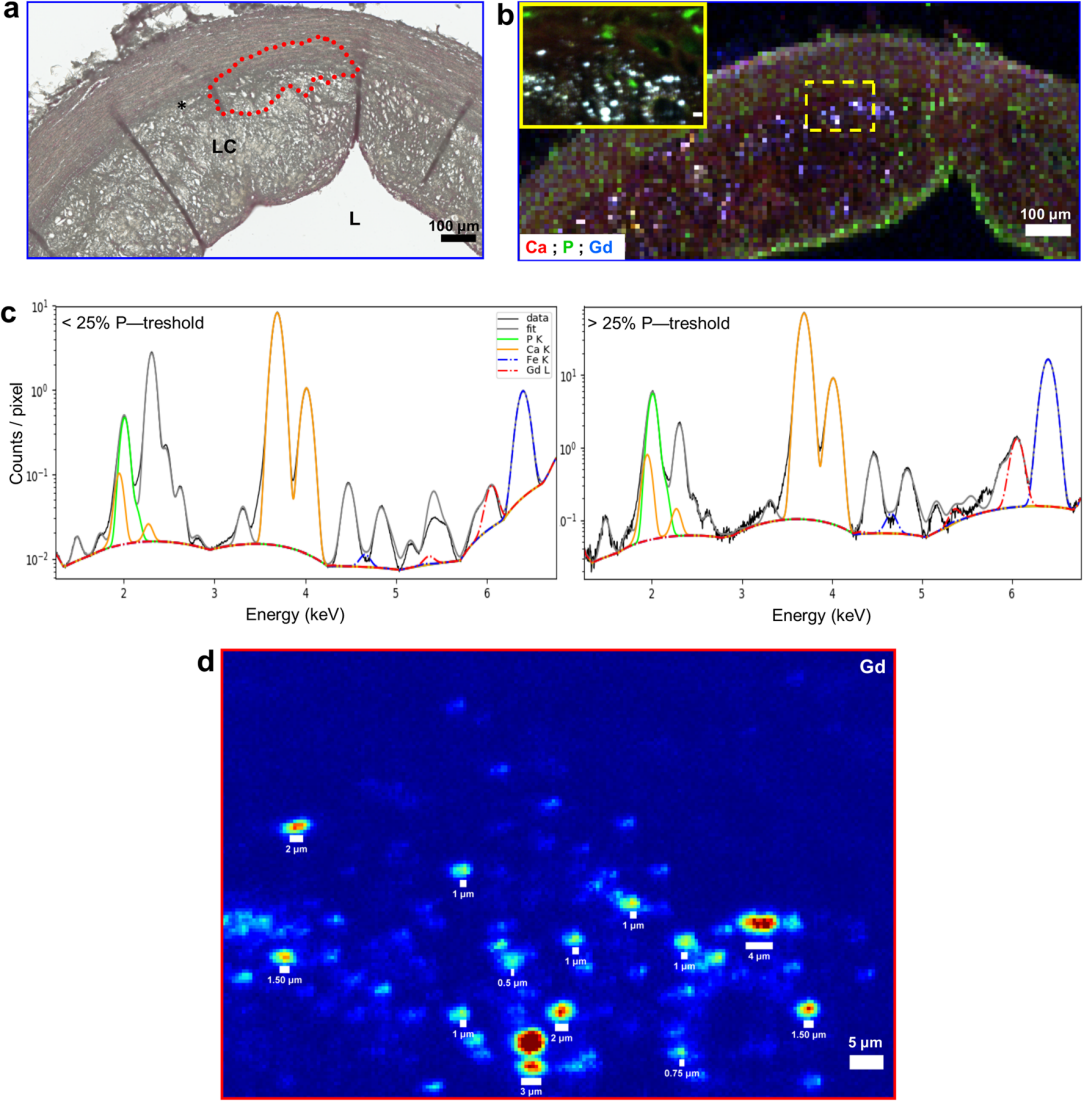
Gd involvement in arterial calcification. **a** Micrograph of a von Kossa-stained advanced plaque ROI (× 15 magnification) reveals arterial calcifications, which are enriched at the intimomedial interface (asterisk, also indicated by the dashed red lines). Scale bar, 100 μm; *L*, lumen; *LC*, lipid core. **b** SR-μXRF analysis and RGB overlay of Ca, P, and Gd distribution at 10-μm and 0.5-μm resolution (ROIs are marked by yellow dashed lines). Scale bars, 100 μm; ROI, 5 μm. **c** Cellular uptake of Gd is investigated by analyzing the P distribution as a marker of cell membrane, ATP, or nucleic acids. P distribution maps at 0.5-μm resolution are segmented into two compartments by applying a 25 % threshold on the maximum of the P K-line fluorescence signal and comparing elemental concentrations in P-poor (< 25 % P-Threshold) or P-rich (> 25 % P-Threshold) areas. Corresponding spectral deconvolution displaying colocalizing elements are normalized by the number of pixel of each region, thus giving an averaged spectrum for each region. The amplitude of the peak corresponding to the respective element is scaled by the amount of atoms being probed by the X-ray beam. In contrast to P-poor areas with < 1 mM, 62 mM, and 87 mM of Gd, P and Ca, respectively, P-rich areas contained > 2 mM, 779 mM, and 789 mM of Gd, P and Ca, respectively. Since P-rich compartments mark the cells or indicate close proximity to the cells, Gd could have been taken up by the cells, presumably by those that undergo calcified apoptosis, or may be involved in extracellular mineralization through complexation with P and Ca. **d** Size distribution analysis shows Gd-rich hotspots ranging from a few micrometers to submicrometers, which corroborates with von Kossa-stained calcified deposits, and indicates Gd involvement in arterial calcification.

SR-μXRF analysis at 30-μm-step width detected Gd in 4 samples—one early lesion and 3 advanced plaques. Predefined ROIs within these specimens were further investigated for their Gd content at higher resolution down to 0.5 μm, which displayed increasing Gd concentrations (Fig. 4). Higher-resolution scans revealed increasing local Gd, P, and Ca concentrations in progressing plaques (Figs. 4 and 5). SR-μXRF analysis at 10-μm- and 2-μm-step width and RGB overlay of P, S, and Fe maps revealed distinct Fe distribution around cell- or collagen-rich areas in the intima (Fig. 6). In comparison, RGB overlay of Gd, S, and Fe maps in addition to P, S, and Gd maps revealed Gd distribution within the deeper areas of the intima, especially along the intimomedial interface at increasing concentrations colocalized with P and Ca. Finally, analysis of possible cellular uptake of Gd (see the “Materials and Methods” section) yielded Gd, P, and Ca concentrations of ~ 0.1 mM, 62 mM, and 87 mM, respectively, in areas below the threshold (Fig. 7, electronic supplementary material, Fig. S5). In areas above the threshold, which indicates elemental hotspots, these concentrations were ~ 2.5 mM, 779 mM, and 789 mM, respectively. Size distribution analysis revealed Gd-rich hotspots ranging from a few micrometers to submicrometers, which corroborated with von Kossa-stained calcified deposits.

## Discussion

The aim of this study was to investigate the microdistribution of Gd- and iron oxide-based CA in the atherosclerotic plaques of NZW rabbits after IV injection of Eu-VSOP and Gd-BOPTA. VSOP are increasingly being investigated in MR imaging of experimental atherosclerosis owing to their early uptake (< 2 h) into atherosclerotic plaques that correlates with accumulation of the ECM rather than phagocytosis [15, 16]. ECM accumulation is a marker of atherosclerotic plaque progression; therefore, correlations of ECM material with Fe concentration, Eu signal intensity as a surrogate marker of VSOP, and Gd concentration were investigated [35]. Eu-doping of the iron oxide cores of the nanoparticles enabled their detection by the LA-ICP-MS analysis and distinguished their distribution from that of endogenous Fe [26]. Overall, our results show correlations between ECM material and Fe concentration or Eu signal intensity.

Published data suggest that VSOP enter atherosclerotic plaques through the endothelium or macrophage phagocytosis [36]. In endothelial cells, uptake was shown to be mediated by endosomal pathway [37]. These findings are supported by our investigations, which demonstrate Eu-VSOP mostly along the endothelium, superficially in the subendothelial space and at the boundaries of the adventitia, media, and intima (Fig. 2). Although Eu detection remained beyond the sensitivity of SR-μXRF, RGB overlays of P, S, and Fe maps highlighted Fe distribution in cell- or collagen-rich areas (Fig. 6). Cells respond to changes in Fe concentration by interactions involving Fe transport, Fe-S clustering or storage proteins, and highly sulfated GAGs [38]. IONP can be metabolized to soluble Fe, which might be stored as ferritin or released into an inflammatory environment [39]. Increased vasa vasorum activity results in the formation of fragile microvessels that might enhance nanoparticle uptake into the plaque ECM (Fig. 1, electronic supplementary material, Fig. S2) [40].

Gd concentration, unlike that of Fe, did not correlate with increased ECM material*.* Remarkably though, compared to healthy arteries, maximum Gd concentrations were 4 and 21 times higher in early and advanced plaques, respectively. This indicates focal Gd hotspots. Advanced plaques were characterized by a large number of such hotspots in deeper regions of the intima corresponding to the intimomedial interface, which hosts large lipid pools and macrophages (Figs. 1 and 3). In ROIs including such areas, SR-μXRF analysis at ~ 0.5-μm resolution confirmed Gd content exceeding mM concentrations with high P and Ca colocalization (Fig. 5). These results are in line with studies suggesting insoluble complex formation [20, 21]. An earlier study suggests that Gd uptake into atherosclerotic plaques occurs through binding and complexation with serum albumin and breakdown of Gd-chelates at the endothelium or vasa vasorum [41]. The authors of this study postulate ECM accumulation of Gd through interactions with collagen, proteoglycans, and tenascin [41]. Another study identified the integrity of the collagen network in cartilage to be a determinant of gadopentetate dimeglumine (Magnevist) accumulation [42]. Our results are consistent with these findings in that dense stretches of collagen fibrils resulted in boundary formation around lipid-rich zones (Fig. 6, electronic supplementary material, Fig. S1). This might lead to selective Gd deposition along the lipid pool-rich intimomedial interface or Gd concentration gradients, possibly through interactions with GAGs. GAGs are upregulated during plaque progression [43]. Their highly negatively charged side chains contribute to complex formation through establishing electrostatic bridges or performing higher level of interactions especially with positive amino-acid residues of proteins including low density lipoprotein (LDL), platelet factor-4 (PF4), or fibroblast growth factor-2 (FGF-2) [44]. This ability makes them excellent candidates for ligand competition reactions, especially through facilitation of highly abundant divalent or trivalent cations, which stabilize these complexes [45]. Although Gd-BOPTA is negatively charged, it is likely to undergo partial or complete chelate dissociation in metabolically active inflammatory sites of the atherosclerotic ECM. Notably, the MRI signal-enhancing effect of Gd was reported to increase dramatically after GBCA incubation including Gd-BOPTA in heparin, which is a highly sulfated GAG type [21].

Finally, although GBCA were originally developed to distribute extracellularly after IV administration, it has been speculated that reactive cellular responses may occur [46]. Inhibition of phagocytosis and macrophage apoptosis upon Gd exposure have been reported [47, 48]. The threshold we applied on the fluorescence signal detected for the P distribution at 0.5-μm resolution analyzed by SR-μXRF corroborates a cellular response since high P distribution marks cell membrane, ATP, or nucleic acids. Increased Gd, P, and Ca concentrations in areas above the threshold confirm Gd hotspots and further support complexation with P and Ca*.* The size distribution of these hotspots ranging from a few micrometers to submicrometers indicates Gd involvement in arterial calcification, presumably through uptake by cells that undergo calcified apoptosis or extracellular mineralization (Fig. 7, electronic supplementary material, Fig. S5) [49]. Arterial calcification is an active and highly regulated process of atherogenesis. It is similar to bone formation, occurs parallel to arterial lipid build-up, and becomes a key characteristic of advanced plaques [50]. We detected such calcifying regions in advanced plaque sections, especially in microzones along the intimomedial interface colocalizing with Gd hotspots (Figs. 1 and 7).

Our study has several limitations including sample processing and the small sample size. Investigation of a larger sample set in this study would have been excessively demanding and not feasible for elemental microscopy during our granted beam time at the European Synchrotron Radiation Facility. Sample processing by means of tissue fixation and paraffin embedding is a standard method in IHC analysis. Paraffin infiltration facilitates uniform slicing and reproducibility of serial sectioning, although it might also result in capture of elements. We performed deparaffinization before LA-ICP-MS analysis; however, ethanol-washing steps during this process might have altered the measured elemental concentrations we measured, particularly for elements involved in transient interactions.

## Conclusion

This is the first study comparing the microdistribution of Gd- and iron oxide-based MR contrast agents in atherosclerotic plaques. We performed IHC and correlative elemental microscopy by LA-ICP-MS and SR-μXRF, and achieved a limit of detection at ~ 0.1 nmol/g and a spatial resolution at 0.5 μm. Overall, these methods allowed differentiation of atherosclerotic plaque tissue distributions of Eu-VSOP and Gd, which are determined by the microstructural ECM composition. We found arterial vessel wall Fe concentration and Eu signal intensity to increase with atherosclerotic plaque progression, suggesting that Eu-VSOP might be used to monitor plaque progression by experimental MRI investigations. Furthermore, we found that the intimomedial interface is a crucial microenvironment for Gd-rich elemental hotspots. Colocalization of increased Ca and P and the size distribution of Gd hotspots ranging from a few micrometers to submicrometers indicate arterial calcification, which might help in characterizing morphological changes underlying plaque vulnerability, and in turn pave the way for MRI signal intensity-based discrimination of stable plaques from vulnerable plaques.

## Supplementary Information

ESM 1 (DOCX 11.4 kb)
