# Supplementary material for: Microdistribution of Magnetic Resonance Imaging Contrast Agents in Atherosclerotic Plaques Determined by LA-ICP-MS and SR-μXRF Imaging
Source: Mol Imaging Biol. 2020 Dec 7;23(3):382–93. doi: 10.1007/s11307-020-01563-z (PMC8099766; doi:10.1007/s11307-020-01563-z)
Supplement: Supplementary file 1 — (DOCX 11.4 kb) [file 11307_2020_1563_MOESM1_ESM.docx]

**Electronic Supplementary Material**

**Title: Microdistribution of Magnetic Resonance Imaging Contrast Agents in Atherosclerotic Plaques Determined by LA‑ICP-MS and SR-µXRF Imaging**

**Journal**: Molecular Imaging and Biology

**Authors: Yavuz Oguz Uca^1^*, David Hallmann^2^, Bernhard Hesse^3,4^, Christian Seim^3,5^, Nicola Stolzenburg^1^, Hubertus Pietsch^2^, Jörg Schnorr^1^, Matthias Taupitz^1^**

**Affiliations**: ^1^ Charité - Universitätsmedizin Berlin, corporate member of Freie Universität Berlin, Humboldt‑Universität zu Berlin, and Berlin Institute of Health, Berlin, Germany, ^2^ MR and CT Contrast Media Research, Bayer AG, Berlin, Germany, ^3^ Xploraytion GmbH, Berlin, Germany, ^4^ European Synchrotron Radiation Facility (ESRF), Grenoble, France, ^5^ Physikalisch‑Technische Bundesanstalt (PTB), Berlin, Germany

**Corresponding author**: * Yavuz Oguz Uca, Charité - Universitätsmedizin Berlin, corporate member of Freie Universität Berlin, Humboldt‑Universität zu Berlin, and Berlin Institute of Health, Charitéplatz 1, 10117 Berlin, Germany. Phone: +49 (30) 450539066

email: [yavuz-oguz.uca@charite.de](mailto:yavuz-oguz.uca@charite.de)

ORCID: Yavuz Oguz Uca: <https://orcid.org/0000-0003-3268-4382>

**MATERIAL AND METHODS**

**Induction of Atherosclerosis**

Twelve male New Zealand White (NZW) rabbits (Charles River Laboratories Germany GmbH, Sulzfeld, Germany) with initial body weights of 1.5 – 2.0 kg were kept individually in conventional cages with access to water and hay briquettes ad libitum. For induction of atherosclerosis, they were fed pelleted complete diet supplemented by 0.2% cholesterol (Altromin Spezialfutter GmbH & Co. KG, Lage, Germany) ad libitum for a total of 26 weeks. At 4 and 8 weeks after the start of the diet, the rabbits were subcutaneously injected with heat shock protein (65 kD HSP from Mycobacterium bovis BCG, fragment 180-188, Sigma‑Aldrich Chemie GmbH, Steinheim, Germany).[1] At 8 weeks after the start of the diet, vascular endothelial growth factor (recombinant human VEGF, Sigma‑Aldrich Chemie GmbH, Steinheim, Germany) was intravenously (IV) injected.[2] Pathologic features defined by the American Heart Association (AHA) were taken into consideration for qualitative and score-based semiquantitative assessment of plaque pathomorphology (Table S1, Fig. S1).

This model of atherosclerosis has been extensively used in advancing molecular imaging modalities, especially magnetic resonance imaging (MRI). Earlier studies have advanced our understanding of the organ and tissue distribution characteristics of MR contrast agents (CA) and their uptake by specialized cells, i.e., macrophages, in atherosclerotic plaques.[3–6] Morphologic features of plaque instability in rabbits are very close to those in humans.[7] The major difference between the NZW rabbit and Watanabe heritable hyperlipidemic (WHHL) rabbit model of atherosclerosis lies in lipid-loading characteristics. While higher loads of lipid pools, e.g., several lipid cores, might be present in the arteries of hyperlipidemic rabbits, vasa vasorum activity originating from the adventitia and extending into the intima, endothelium and microvessels have important roles in the development of atherosclerosis in the NZW rabbit model (Fig. S2).[8]

Table S1: Pathologic features of atherosclerotic plaque progression and American Health Association (AHA) classification.[9, 10]

| AHA classification | Pathomorphologic characteristics |
| --- | --- |
| Early  (type I-III) | - Initial lesion: low level of extracellular matrix (ECM) accumulation with occasional fatty streaks, no or minimal level of macrophages. - Intact and thick media with contractile smooth muscle cell (SMC) phenotype. - Early lesion prone to progression into intermediate plaque. (Type III=preatheroma). |
| Advanced  (type IV-V) | - Increasing lipid and ECM accumulation. - Increasing macrophage content in the intima, foam cell formation, apoptosis. Lipid cores are detectable. Fibrous cap covers lipid cores. - Medial thinning, synthetic SMC migration into the intima. - Appearance of atheroma in the form of fibrous atheroma, calcific atheroma or fibrotic atheroma. |
| Advanced  (type VI -) | - Increased ECM degradation and remodeling. - Enlarging lipid cores, necrotic core formation. Thinning of fibrous cap with increasing matrix-metalloproteinase activity. - Fibrous, calcified or fibrotic atheroma transforms into vulnerable plaque. - Hematoma/hemorrhage and/or thrombotic deposit. |

**Elemental Microscopy**

In this study, LA-ICP-MS analysis results were stored as datasets in csv format. Each dataset consisted of multiple data lines of the same tissue material analyzed by continuous‑line ablation. For generation of 2D elemental images, full dataset of a specific specimen was imported into the MassImager, a free software developed by Robin Schmidt.[11] Predefined parameters of the analysis such as the laser spot size and scanning speed were set accordingly to generate an image. Arterial vessel wall (media, intima and endothelium) elemental concentrations were quantified following matrix‑matched calibration (Fig. S3). For that, matrix‑matched laboratory standards of well‑defined element concentrations were spiked onto gelatin and scanned. By using the ‘freehand’ detection tool, the arterial vessel area was distinguished.Regions of interest (ROIs) were drawn to calculate relative signal intensity recorded in counts per second (CPS). Mean signal intensity values were extracted and transferred to Microsoft Office Excel, and regression formula was calculated. This formula was inserted back into the respective panel in the MassImager software, and histogram analysis was performed to determine data distribution, standard deviation, minima, and maxima. To reduce the background noise signal for iron (Fe), europium (Eu), and gadolinium (Gd), minima were set to 99.0 nmol/g, 49.5 (CPS), and 0.5 nmol/g, respectively, and maxima were set to 2%, 1%m and 1%, respectively. Concentration values and the standard deviations were transferred back to Microsoft Office Excel, where they were categorized under the respective lesion type (control, early lesion and advanced plaque). Mean concentration values and their respective standard deviations were calculated, concentration graphs were drawn, and error bars were generated. Due to the advanced intrinsic sensitivity of the method and matrix-matched calibration, quantification of the arterial vessel wall Gd and Fe concentrations was only performed on the LA‑ICP‑MS results.

Synchrotron radiation μX-ray fluorescence (SR-μXRF) investigations at the ID21 beamline at the European Synchrotron Radiation Facility (ESRF) in Grenoble, France were performed according to the routine applications developed and implemented by the Beamline Instrumentation Software Support (BLISS) group at the ESRF. XRF spectroscopy analysis (XRF spectroscopy normalization, spectral deconvolution, generation of 2D elemental distribution maps, and quantification) was done using PyMCA (Python multichannel analyzer), the open source software developed at the facility (Fig. S4).[12] PyMCA implements most of the needs of XRF spectroscopy, and extracts elemental concentrations in terms of mass fractions or molar concentrations based on certain matrix determination. We have assumed the matrix composition of the arterial tissue specimens according to the published data.[13] The geometry and the flux at the XRF setup are known, and were used as input parameter in PyMCA, which was then considered to compute the concentrations. A limitation here is that the same matrix composition as in Veronesi et. al. was assumed all over the samples, and the drying process was neglected to have significant impact on the mass fractions of the remaining material. The manifestation of atherosclerosis is highly heterogeneous, which adds additional complexity to the determination of the matrix composition of the arterial specimens analyzed. Therefore, this was a simplification, thus the reported molar concentrations or mass fractions derived from XRF are rather semi-quantitative. Only predefined ROIs based on the immunohistochemistry (IHC) were quantified by SR-XRF analysis. For that, ‘freehand’ detection tool was used to distinguish the ROI area. Molar concentrations of Gd, Fe, phosphorus (P), and calcium (Ca) at spatial resolutions of 10 μm, 1-2 μm, or 0.5 μm depending on the size of the ROI and the measured XRF spectrum in each point of the image were extracted. These values were transferred to Microsoft Office Excel, where they were categorized under the respective lesion type (control, early lesion and advanced plaque). Mean molar concentrations were calculated, and concentration graphs were generated. Cellular uptake of Gd was investigated by analyzing P distribution as a marker of cell membrane, ATP, or nucleic acids (Fig. S5). XRF spectra of advanced plaque sections at 0.5 µm resolution were analyzed using a 25% threshold. In areas with fluorescence below and above the threshold were revaluated and concentrations were compared. Size distribution analysis of Gd hotspots was done using ImageJ software.

**DISCUSSION**

The aim of this study was to investigate the microdistribution of Gd- and iron oxide-based CA in the atherosclerotic plaques of NZW rabbits after IV injection of Eu-VSOP and Gd‑BOPTA. VSOP are increasingly being investigated in MR imaging of experimental atherosclerosis owing to their early uptake (<2 h) into atherosclerotic plaques that correlates with accumulation of the ECM rather than phagocytosis.[14, 15] On the other hand, Gd-BOPTA was often preferred for vascular MRI studies at the time of the previous in vivo MRI study. Currently, it is only used in liver imaging due to a weak selective signal intensity enhancement of the liver.

Atherosclerosis-free control specimens (Eu-VSOP-negative and Gd-positive) were Vasovist (Gadofosveset trisodium) and elastin-specific GBCA.[16] Quantification results using LA‑ICP‑MS revealed Gd concentration of 2.22 nmol/g (±0.14 nmol/g) in the control group. However, Gd concentrations for the 2 individual control sections that received elastin-specific GBCA or Vasovist were 3.70 nmol/g (±0,14 nmol/g) and 0.73 nmol/g (± 0,14 nmol/g), respectively. These findings are meaningful in that healthy arteries are composed of stable elastic lamella; therefore, a higher Gd concentration for elastin-specific was expected. On the other hand, Vasovist is a non-specific formulation of a stable gadolinium diethylenetriaminepentaacetic acid (Gd-DTPA) chelate derivative. Gd in non-specific GBCA could be washed out during sample preparation procedures unless it interacts with other molecules or elements and becomes insoluble. Therefore, less Gd concentration detected for this control specimen is also meaningful. Although our control sample size was small, the use of elastin‑specific GBCA and Vasovist provided an internal confirmation for our quantification findings.

Elemental analysis has emerged as a sophisticated analytical approach to complement MRI studies with improved tissue detection ability of CAs.[17] It has received valuable attention following reports on tissue retention of Gd initially in nephrogenic systemic fibrosis (NSF).[18] Of such techniques, mass spectrometry (MS) imaging offers the highest limit of detection (LOD) owing to specific mass-to-charge ratios (m/z) of elements.[19] Matrix-assisted laser desorption ionization imaging (MALDI‑MSI) is the most well‑established and widely used method for mapping elements and small biomolecules on histological specimen.[20] Secondary ion MS (SIMS) with time of flight (TOF) mode offers similar sensitivity with submicrometer resolution.[21] Matrix‑associated interferences in MALDI-MS and special sample preparation required for SIMS have been addressed by LA‑ICP-MS.[22] LA-ICP-MS offers great sensitivity and reliable quantification by matrix‑matched calibration methods.[23] The method is applicable for both formalin or cryo-fixed tissues and has been increasingly reported as a fast and a suitable method to complement MRI.[24–26] The major drawback is the spatial resolution that is typically around tens of micrometers. In contrast, XRF analysis offers spatial resolution at submicrometer level. Hard X-ray’s large penetration depth and absence of bremsstrahlung allow for non-destructive investigation on cellular and even subcellular level.[27, 28] From various X-ray methods utilizing different beam sources, SR-µXRF offers up to ten nanometer resolution, and is currently the only method available for quantitative imaging of whole cells.[29] For the reasons explained here, we performed our elemental microscopy investigations by LA‑ICP‑MS, and SR‑µXRF spectroscopy.

**REFERENCES**

1. Xu Qingbo (2002) Role of Heat Shock Proteins in Atherosclerosis. Arterioscler Thromb Vasc Biol 22:1547–1559.

2. Celletti FL, Waugh JM, Amabile PG, et al (2001) Vascular endothelial growth factor enhances atherosclerotic plaque progression. Nat Med 7:425–429.

3. Kooi ME, Cappendijk VC, Cleutjens KBJM, et al (2003) Accumulation of Ultrasmall Superparamagnetic Particles of Iron Oxide in Human Atherosclerotic Plaques Can Be Detected by In Vivo Magnetic Resonance Imaging. Circulation 107:2453–2458.

4. Amirbekian V, Lipinski MJ, Briley-Saebo KC, et al (2007) Detecting and assessing macrophages in vivo to evaluate atherosclerosis noninvasively using molecular MRI. Proc Natl Acad Sci 104:961–966.

5. Tang TY, Muller KH, Graves MJ, et al (2009) Iron Oxide Particles for Atheroma Imaging. Arterioscler Thromb Vasc Biol 29:1001–1008.

6. Hyafil F, Vucic E, Cornily J-C, et al (2011) Monitoring of arterial wall remodelling in atherosclerotic rabbits with a magnetic resonance imaging contrast agent binding to matrix metalloproteinases. Eur Heart J 32:1561–1571.

7. Libby P (2001) What have we learned about the biology of atherosclerosis? The role of inflammation. Am J Cardiol 88:3–6. https://doi.org/10.1016/S0002-9149(01)01879-3

8. Carlier S, Kakadiaris IA, Dib N, et al (2005) Vasa vasorum imaging: A new window to the clinical detection of vulnerable atherosclerotic plaques. Curr Atheroscler Rep 7:164–169.

9. Stary HC (2000) Natural History and Histological Classification of Atherosclerotic Lesions: An Update. Arterioscler Thromb Vasc Biol 20:1177–1178. https://doi.org/10.1161/01.ATV.20.5.1177

10. Virmani R, Kolodgie FD, Burke AP, et al (2000) Lessons From Sudden Coronary Death: A Comprehensive Morphological Classification Scheme for Atherosclerotic Lesions. Arterioscler Thromb Vasc Biol 20:1262–1275.

11. Radbruch A, Richter H, Fingerhut S, et al (2019) Gadolinium Deposition in the Brain in a Large Animal Model: Comparison of Linear and Macrocyclic Gadolinium-Based Contrast Agents. Invest Radiol 54:531.

12. Solé VA, Papillon E, Cotte M, et al (2007) A multiplatform code for the analysis of energy-dispersive X-ray fluorescence spectra. Spectrochim Acta Part B At Spectrosc 62:63–68.

13. Veronesi G, Deniaud A, Gallon T, et al (2016) Visualization, quantification and coordination of Ag + ions released from silver nanoparticles in hepatocytes. Nanoscale 8:17012–17021.

14. Ludwig A, Poller WC, Westphal K, et al (2013) Rapid binding of electrostatically stabilized iron oxide nanoparticles to THP-1 monocytic cells via interaction with glycosaminoglycans. Basic Res Cardiol 108:328.

15. Wagner S, Schnorr J, Ludwig A, et al (2013) Contrast-enhanced MR imaging of atherosclerosis using citrate-coated superparamagnetic iron oxide nanoparticles: calcifying microvesicles as imaging target for plaque characterization. Int J Nanomedicine 8:767–779.

16. Makowski MR, Wiethoff AJ, Blume U, et al (2011) Assessment of atherosclerotic plaque burden with an elastin-specific magnetic resonance contrast agent. Nat Med 17:383–388.

17. Pugh JAT, Cox AG, McLeod CW, et al (2012) Elemental imaging of MRI contrast agents: benchmarking of LA-ICP-MS to MRI. Anal Bioanal Chem 403:1641–1649.

18. Grobner T (2006) Gadolinium – a specific trigger for the development of nephrogenic fibrosing dermopathy and nephrogenic systemic fibrosis? Nephrol Dial Transplant 21:1104–1108.

19. Becker JS, Matusch A, Wu B (2014) Bioimaging mass spectrometry of trace elements – recent advance and applications of LA-ICP-MS: A review. Anal Chim Acta 835:1–18.

20. Acquadro E, Cabella C, Ghiani S, et al (2009) Matrix-Assisted Laser Desorption Ionization Imaging Mass Spectrometry Detection of a Magnetic Resonance Imaging Contrast Agent in Mouse Liver. Anal Chem 81:2779–2784.

21. Abraham JL, Chandra S, Thakral C, et al (2008) SIMS imaging of gadolinium isotopes in tissue from Nephrogenic Systemic Fibrosis patients: Release of free Gd from magnetic resonance imaging (MRI) contrast agents. Appl Surf Sci 255:1181–1184.

22. Becker JS, Zoriy M, Becker JS, et al (2007) Laser ablation inductively coupled plasma mass spectrometry (LA-ICP-MS) in elemental imaging of biological tissues and in proteomics. J Anal At Spectrom 22:736.

23. Becker JS, Zoriy MV, Pickhardt C, et al (2005) Imaging of Copper, Zinc, and Other Elements in Thin Section of Human Brain Samples (Hippocampus) by Laser Ablation Inductively Coupled Plasma Mass Spectrometry. Anal Chem 77:3208–3216.

24. Sussulini A, Wiener E, Marnitz T, et al (2013) Quantitative imaging of the tissue contrast agent [Gd(DTPA)]2− in articular cartilage by laser ablation inductively coupled plasma mass spectrometry. Contrast Media Mol Imaging 8:204–209.

25. Wang HA, Grolimund D, Giesen C, et al (2013) Fast chemical imaging at high spatial resolution by laser ablation inductively coupled plasma mass spectrometry. Anal Chem 85:10107–10116

26. Trunova V, Sidorina A, Zvereva V, et al (2013) Changes in the elemental content of rat heart as a result of the fixation in formalin analyzed by synchrotron radiation X-ray fluorescent analysis. J Trace Elem Med Biol 27:76–77.

27. Paunesku T, Vogt S, Maser J, et al (2006) X-ray fluorescence microprobe imaging in biology and medicine. J Cell Biochem 99:1489–1502.

28. Thakral C, Abraham JL (2007) Automated Scanning Electron Microscopy and X-Ray Microanalysis for in situ Quantification of Gadolinium Deposits in Skin. J Electron Microsc (Tokyo) 56:181–187.

29. Gramaccioni C, Yang Y, Procopio A, et al (2018) Nanoscale quantification of intracellular element concentration by X-ray fluorescence microscopy combined with X-ray phase contrast nanotomography. Appl Phys Lett 112:053701


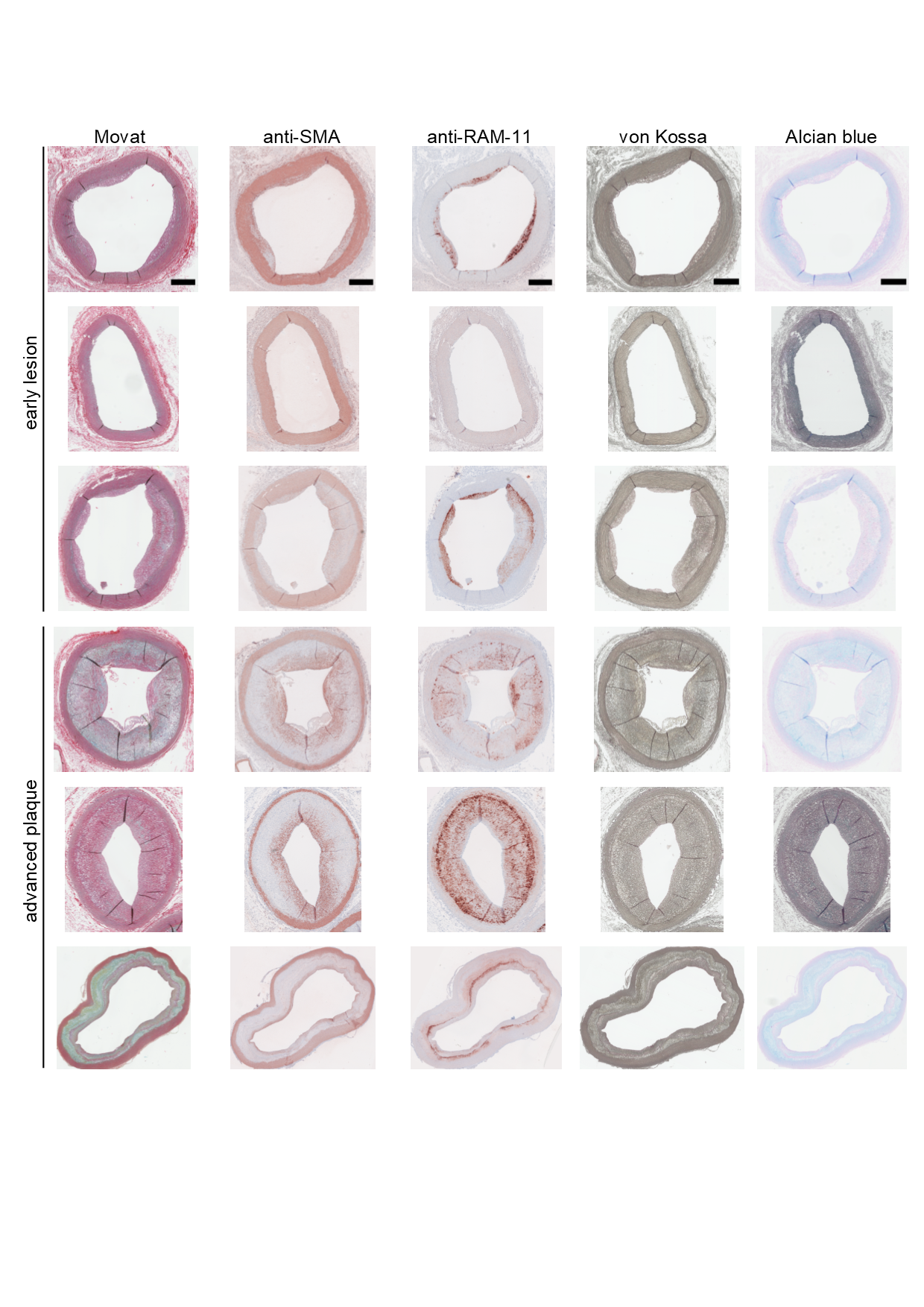

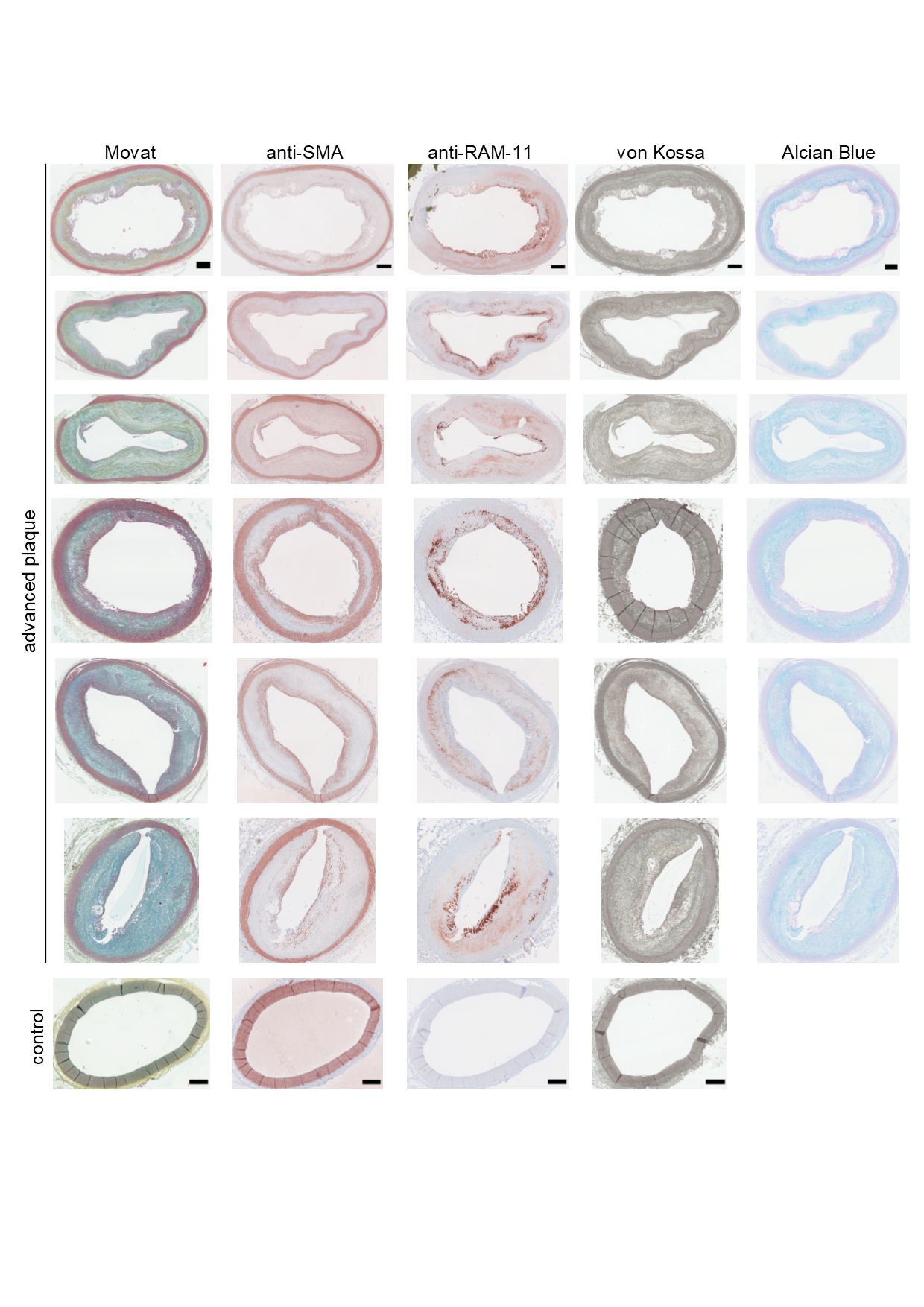


**Figure S1:** Atherosclerotic plaque characterization by immunohistochemistry (IHC). Movat’s staining reveals cells by red staining that are also depicted by anti‑smooth muscle cell (SMC) actin (anti‑SMA) and anti-rabbit macrophage (anti‑RAM‑11) immunostaining. Contractile SMCs in the media and synthetic SMCs migrating into the intima are distinguished by their spindle-like and circular shapes, respectively. Lipid pools are non-stained circular, cleft- or vacuole-like shapes at the intimomedial areas associated with increased macrophage colocalization, indicating foam cell or lipid cores. Lipid pools are surrounded by light blue‑stained glycosaminoglycans (GAG). Von Kossa staining identifies calcifications in the intima, especially along the intimomedial interface of advanced plaques. Scale bars: 500 µm, L: lumen, E: endothelium, I: intima, LC: lipid core, asterisk: intimomedial interface, M: media, A: adventitia, V: vasa vasorum.


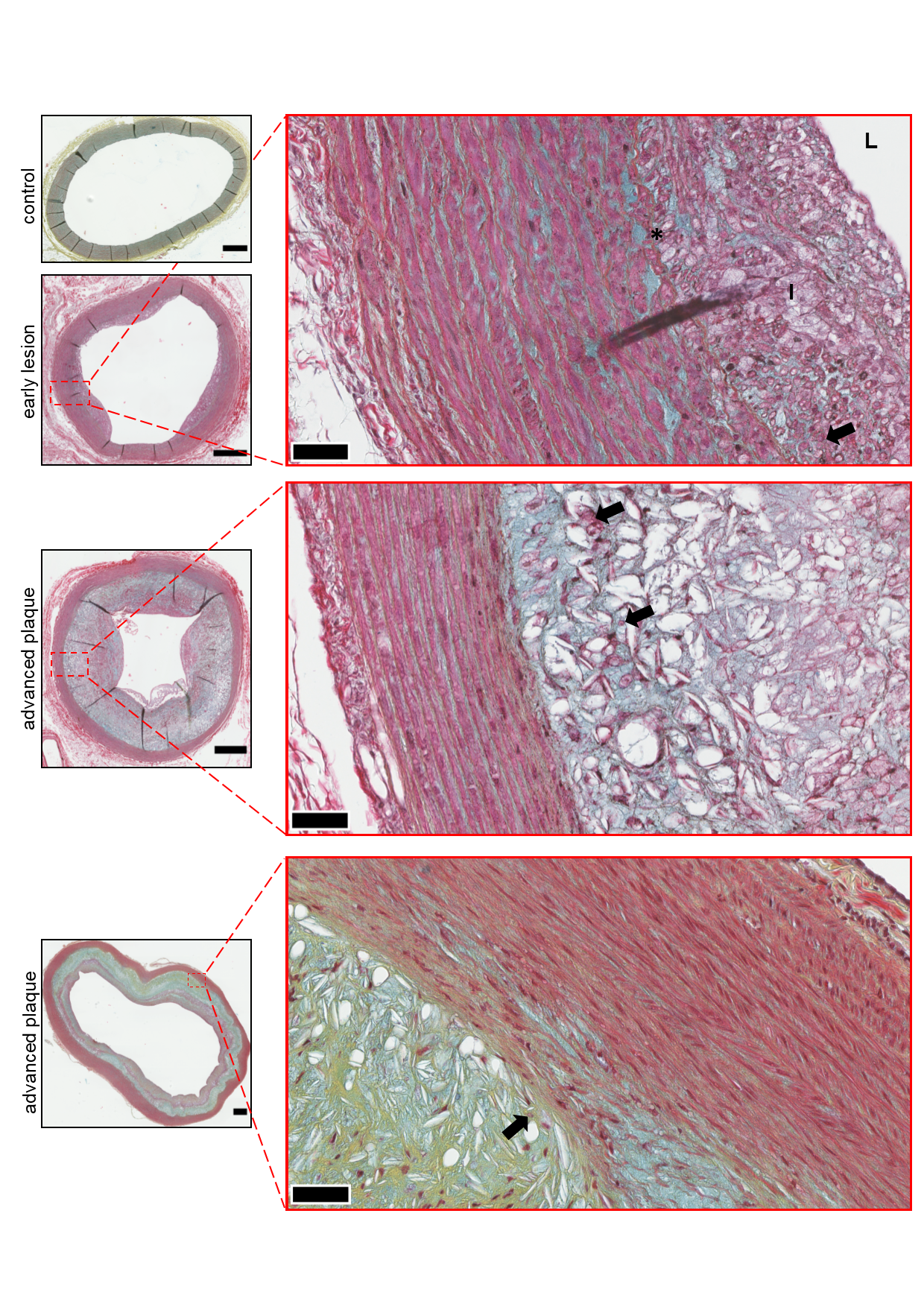


**Figure S2:** Microvessels originating from vasa vasorum and extending into the intimomedial interface. Micrographs of Movat’s stained arterial sections. Region of interest (ROI) (40X magnification), L: lumen, I: intima, asterisk: intimomedial interface, arrows: erythrocytes. Scale bars: 500 µm, ROI: 50 µm.


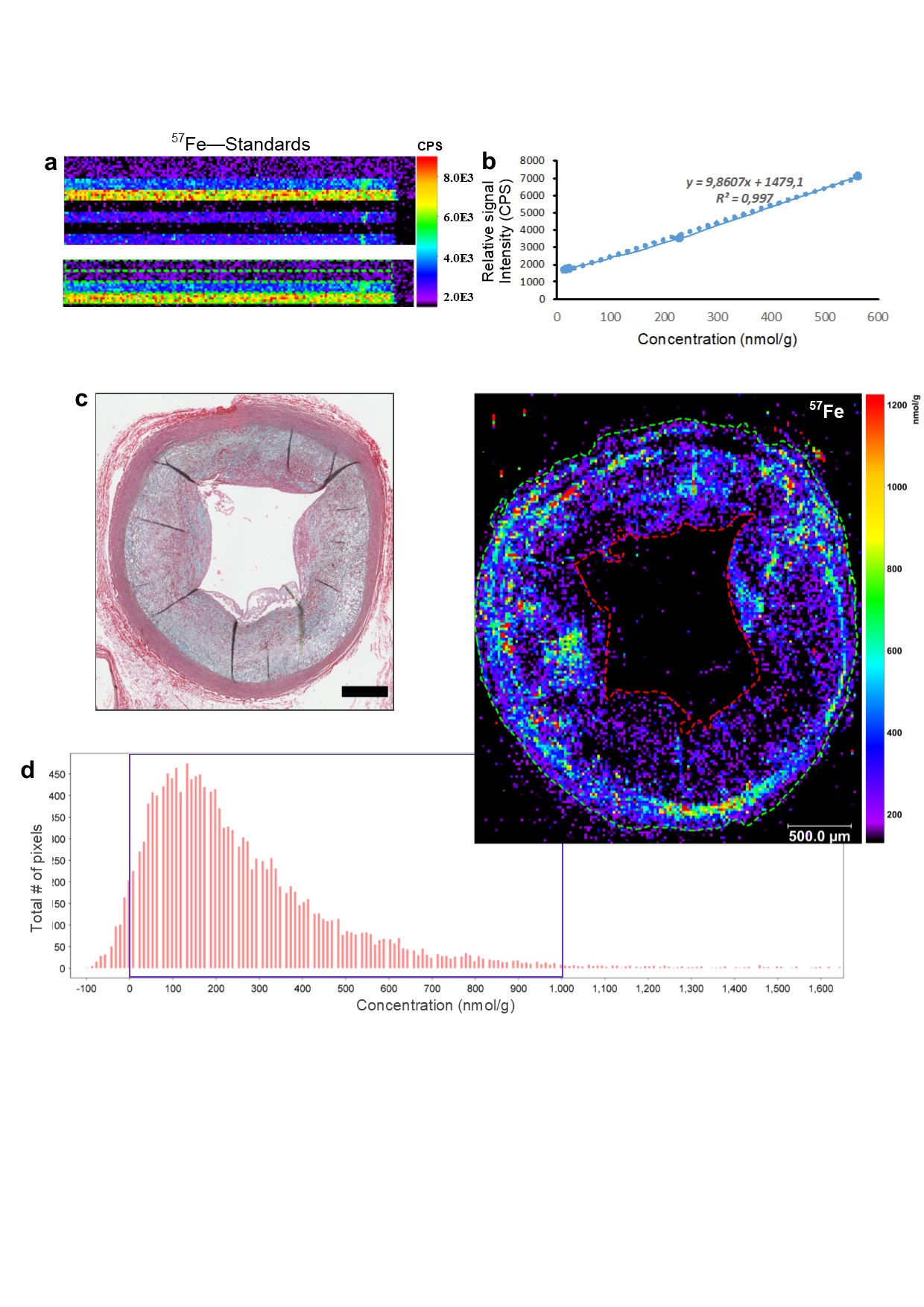


**Figure S3:** Matrix-matched calibration and Iron (Fe) quantification by laser ablation inductively coupled plasma mass spectrometry (LA-ICP-MS). (**a**) 14.7 nmol/g, 22.0 nmol/g, 228.9 nmol/g, and 562.7 nmol/g concentrations of Fe were spiked onto gelatin and scanned. ROIs are drawn to calculate relative signal intensity recorded in counts per second (CPS). (**b**) Regression formula is retrieved to quantify arterial vessel wall (media, intima and endothelium) concentrations. (**c**) Movat’s-stained micrographs are used to define the plaque boundaries. ‘Freehand’ detection tool is used to distinguish the vessel area (green dotted line) excluding the lumen (red dotted line). (**d**) Histogram analysis is performed to determine data distribution, standard deviation, minima and maxima. Scale bars: 500 μm.


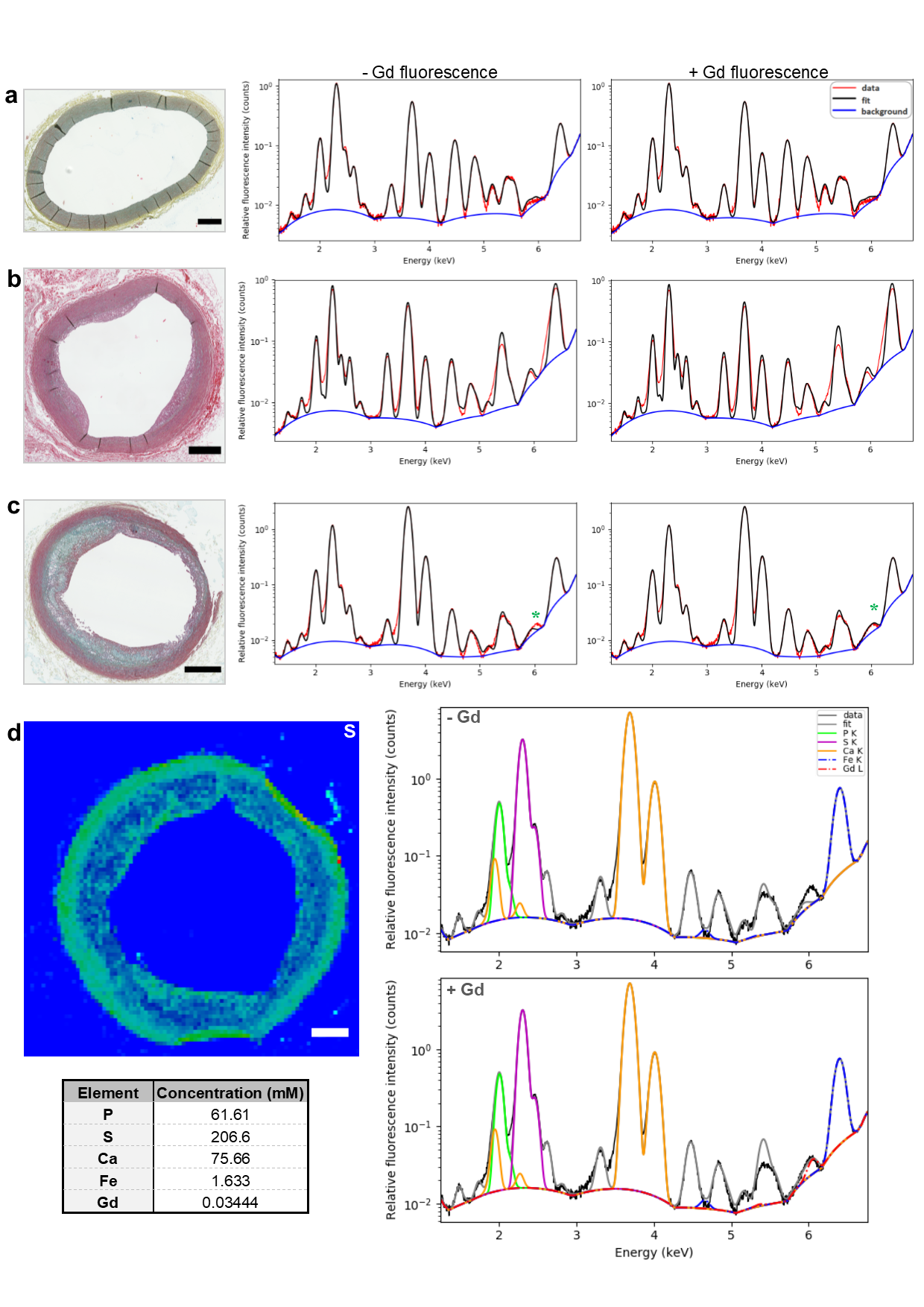


**Figure S4:** Synchrotron radiation µX‑ray fluorescence (SR-µXRF) spectroscopy analysis, and quantification by PyMCA. (**a-c**) Spectral deconvolution displaying the data and the fit overlay in the absence (*left*) or presence (*right*) of characteristic Gd L-line fluorescence in healthy artery (**a**), early lesion (**b**), and advanced plaque (**c**). Movat’s-stained micrographs are provided for comparison. The fit in the absence or presence of Gd fluorescence remains unchanged when the respective element content is beyond the limit of detection. Asterisk (*green*) indicates the change in the fit, confirming Gd detection. (**d**) On the Gd‑detected sections, by taking sulfur (S) distribution maps to define the arterial boundary, XRF spectra are obtained and further analyzed to determine colocalizing elements. Scale bar: 500 μm


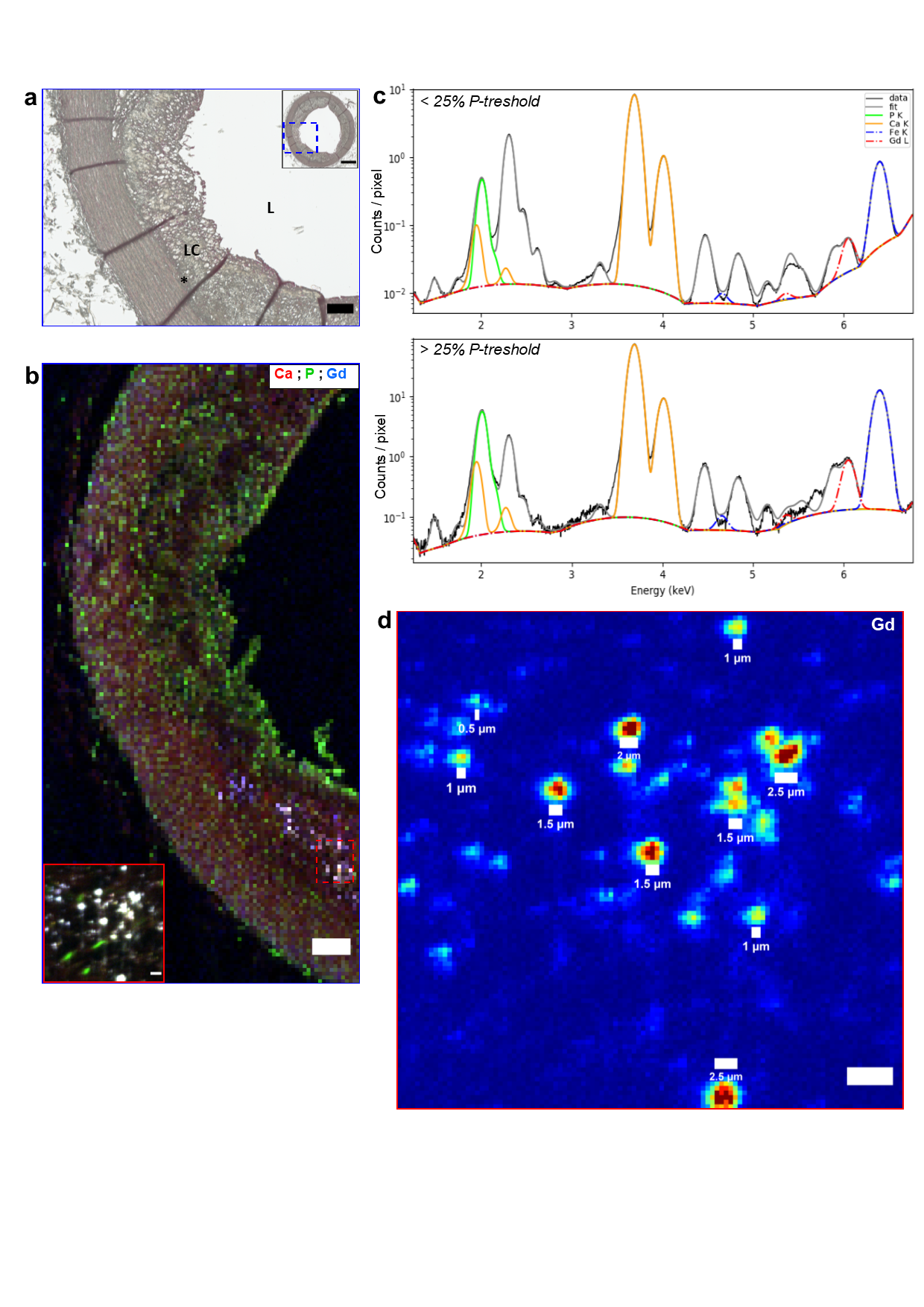


**Figure S5:** Gd involvement in arterial calcification. (**a**) Micrograph of a von Kossa‑stained advanced plaque ROI (15X magnification) reveals arterial calcifications, which are enriched at the intimomedial interface. Scale bars: 500 µm, ROI: 100 µm. (**b**) SR‑µXRF microscopy and ROI RGB overlays of calcium (Ca), phosphorus (P), and Gd distribution at 10 µm and 0.5 µm resolution. Scale bars: 100 µm, ROI: 5 µm. (**c**) Cellular uptake of Gd is investigated by analyzing the P distribution as a marker of cell membrane, ATP, or nucleic acids. P distribution maps at 0.5 µm resolution are segmented into two compartments by applying a 25% threshold on the maximum of the P K-line fluorescence signal and comparing elemental concentrations in P-poor (< 25% P - Threshold) or P‑rich (> 25% P - Threshold) areas. Corresponding spectral deconvolution displaying co‑localizing elements are normalized by the number of pixel of each region, thus giving an averaged spectrum for each region. The amplitude of the peak corresponding to the respective element is scaled by the amount of atoms being probed by the X-ray beam. In contrast to P-poor areas with <1 mM, 62 mM and 87 mM of Gd, P and Ca, respectively, P-rich areas contained >2 mM, 779 mM and 789 mM of Gd, P and Ca, respectively. Since P-rich compartments mark the cells or indicate close proximity to the cells, Gd could have been taken up by the cells, presumably by those that undergo calcified apoptosis, or may be involved in extracellular mineralization through complexation with P and Ca. (**d**) Size distribution analysis shows Gd-rich hotspots ranging from a few micrometers to submicrometers, which corroborates with von Kossa stained calcified deposits, and indicates Gd involvement in arterial calcification. Scale bar: 5 µm. L: lumen, LC: lipid core, asterisk: intimomedial interface.
